# Key competencies of physical education and exercise-related positive wellbeing among secondary school students: indirect pathways through physical self-perception and autonomous motivation

**DOI:** 10.3389/fpsyg.2026.1902000

**Published:** 2026-09-17

**Authors:** Jiawei Wang, Dachao Liu

**Affiliations:** 1 Department of Physical Education and Training, Capital University of Physical Education and Sports, Beijing, China; 2 School of Physical Education and Sport Science, Fujian Normal University, Fujian, China

**Keywords:** autonomous motivation, exercise-related positive well-being, key competencies of physical education, physical self-perception, structural equation modeling

## Abstract

**Purpose:**

This study examined the association between key competencies of physical education (KCPE) and exercise-related positive wellbeing among secondary school students from selected districts of Beijing. It also estimated specific indirect associations through physical self-perception and autonomous motivation, including the specified serial indirect pathway.

**Methods:**

An analytical cross-sectional survey was conducted using stratified cluster sampling among secondary school students from selected districts of Beijing. Participants completed standardized self-report measures, and the data were analyzed using IBM SPSS Statistics 27.0 and AMOS 28.0.

**Results:**

The hypothesized model showed good fit (χ^2^/df = 1.399, CFI = 0.997, TLI = 0.996, RMSEA = 0.021, SRMR = 0.013). KCPE was positively associated with physical self-perception (β = 0.390), autonomous motivation (β = 0.353), and exercise-related positive wellbeing (β = 0.190; all *p* < 0.001). Physical self-perception was positively associated with autonomous motivation (β = 0.280) and exercise-related positive well-being (β = 0.134), and autonomous motivation was positively associated with exercise-related positive wellbeing (β = 0.312; all *p* < 0.001). The standardized indirect associations through physical self-perception [0.052, 95% CI (0.024, 0.081)], autonomous motivation [0.110, 95% CI (0.078, 0.146)], and the specified serial indirect pathway involving both variables [0.034, 95% CI (0.022, 0.048)] were statistically significant. In supplementary models, none of the paths from KCPE, physical self-perception, or autonomous motivation to psychological distress or fatigue was statistically significant.

**Conclusion:**

Among the surveyed students, KCPE was positively associated with exercise-related positive well-being, and physical self-perception and autonomous motivation showed statistically significant indirect associations within this relationship. These cross-sectional findings are consistent with the proposed association pattern but do not confirm temporal precedence, which requires longitudinal evidence.

## 1 Introduction

Adolescents' psychological wellbeing has become an important public health concern worldwide. The World Health Organization (2025)^1^ reports that one in seven adolescents experiences a mental health condition, highlighting the need to identify factors that support positive psychological development during adolescence. Within this context, physical education provides an important developmental setting where students engage in physical activity, interact with others, and develop perceptions of competence. Students' experiences during participation are shaped not only by behavioral engagement but also by affective responses and psychological perceptions formed within the learning environment. Positive exercise-related experiences may support continued participation and the development of favorable exercise habits. Therefore, understanding how competencies developed through physical education relate to students' exercise-related experiences represents an important issue in school physical education research. According to self-determination theory, environments that support autonomy, competence, and relatedness are associated with more adaptive motivational processes and positive affective experiences (White et al., 2021), suggesting that competency development in physical education may be linked to exercise-related experiences through theoretically proposed psychological associations.

The present study focuses on key competencies of physical education (KCPE), which refers to the integrated qualities and abilities that students develop through sustained physical education learning and apply in physical activity, sports participation, and health-related contexts. Within the Chinese physical education curriculum, KCPE comprises three interrelated dimensions: athletic ability, health behavior, and sports morality (He et al., 2022). This competency-oriented framework emphasizes not only the acquisition of physical skills but also the development of health-related behaviors, social responsibility, and the ability to engage effectively in physical activity contexts.

Although KCPE is conceptually related to physical literacy, the two constructs are not interchangeable. Physical literacy is generally regarded as a broad lifelong construct involving the motivation, confidence, physical competence, knowledge, and understanding that enable individuals to engage in physical activity (Grauduszus et al., 2023). In contrast, KCPE is a curriculum-specific construct embedded within the Chinese school physical education system and reflects the intended learning outcomes of physical education, including athletic ability, health behavior, and sports morality. Thus, physical literacy emphasizes an individual's lifelong engagement with physical activity, whereas KCPE emphasizes competency development achieved through formal physical education. However, existing evidence on physical literacy cannot fully explain how curriculum-specific competencies developed within formal physical education relate to students' exercise-related experiences, particularly in school contexts where competency development is embedded within curriculum objectives and instructional practices. Accordingly, KCPE is used as the central educational construct in the present study. Importantly, KCPE represents educational competencies developed through physical education learning rather than students' psychological evaluations of themselves.

Students' experiences in physical education may be associated with their perceptions of competence and reasons for participation, which may influence their subsequent exercise experiences. This possibility highlights the need to examine potential psychological pathways linking KCPE with exercise-related experiences. Athletic ability may be theoretically related to competence-related experiences, health behavior may facilitate self-regulation and informed decision-making, and sports morality may reflect interpersonal experiences relevant to participation contexts. Although these dimensions do not correspond exclusively to individual psychological needs, together they may create supportive conditions that allow students to experience physical activity more positively (Hu et al., 2024).

Physical self-perception represents one potential psychological factor associated with the relationship between KCPE and exercise-related experiences. Based on the Physical Self-Perception Profile (PSPP), physical self-perception refers to individuals' evaluations of their physical abilities and characteristics, including sport competence, physical condition, body attractiveness, physical fitness, and physical self-worth (Fox and Corbin, 1989). Unlike narrower appearance-related constructs such as body image and body esteem (Slade, 1994; Mendelson et al., 2001), physical self-perception represents a broader multidimensional evaluation of the physical self. Among these dimensions, physical self-worth reflects a global evaluation of one's physical self while remaining one component of the broader construct. Students with more favorable physical self-perceptions across competence, condition, fitness, and self-worth domains may report more favorable exercise-related experiences. Previous research has shown that favorable physical self-perceptions are associated with greater motivation and participation in physical activity contexts (Núñez et al., 2021), suggesting that physical self-perception may represent an important psychological pathway associated with the relationship between KCPE and exercise experiences.

Autonomous motivation may represent another important psychological pathway. Within self-determination theory (Deci and Ryan, 2000; Ryan and Deci, 2017), autonomous motivation reflects participation based on personal value, self-endorsement, and enjoyment. Identified regulation, integrated regulation, and intrinsic motivation represent more self-determined forms of exercise regulation, as they involve greater internalization and volition. In contrast, less self-determined forms of regulation are characterized by external pressures or lack of intention. Students with more favorable physical self-perceptions may be more likely to endorse more autonomous reasons for exercise participation. Previous research has demonstrated associations among physical self-perception, autonomous motivation, and sustained physical activity participation (Pulido et al., 2021; Valero-Valenzuela et al., 2021). Therefore, autonomous motivation may provide an additional pathway through which KCPE is associated with students' exercise-related experiences.

However, limited research has examined how these constructs are simultaneously considered within an integrated model among secondary school students. In particular, the psychological processes through which competency development in physical education may relate to students' exercise-related experiences remain insufficiently understood. Building on self-determination theory and previous empirical findings, the present study employed structural equation modeling (SEM) to examine the association between KCPE and exercise-related experiences, with physical self-perception and autonomous motivation proposed as sequential psychological pathways. Given that exercise experiences involve multiple affective dimensions, both favorable and unfavorable aspects of students' exercise experiences were considered. Therefore, this study aimed to examine whether KCPE was associated with exercise-related positive wellbeing and whether physical self-perception and autonomous motivation represented statistically significant indirect associations within this relationship. The following hypotheses were tested:

H1. KCPE is positively associated with exercise-related positive wellbeing.
H2. The indirect association between KCPE and exercise-related positive wellbeing through physical self-perception is statistically significant.
H3. The indirect association between KCPE and exercise-related positive wellbeing through autonomous motivation is statistically significant.
H4. The serial indirect association between KCPE and exercise-related positive wellbeing through physical self-perception and autonomous motivation is statistically significant.

## 2 Materials and methods

### 2.1 Participants

This survey was conducted among secondary school students in Grades 7–9 from selected districts of Beijing, China. Students were recruited using stratified cluster sampling from selected districts of Beijing. The sample included students from 12 secondary schools across 8 districts, involving 36 intact classes. Students were stratified by grade level and district, and participants were recruited from intact teaching groups within each stratum. A total of 1,100 students were invited to participate. Eligibility criteria were: (1) enrollment in Grades 7–9 at a participating school; (2) ability to understand and complete the questionnaire independently; and (3) voluntary agreement to participate. Questionnaires were excluded if substantial missing data or clearly repetitive/invariant response patterns were identified during data screening. With assistance from physical education and homeroom teachers, questionnaires were administered to the selected participants. Of the 1,100 questionnaires distributed, 921 valid responses were retained; 179 were excluded because of missing data or clearly repetitive response patterns, yielding a valid response rate of 83.7%. Participant characteristics are shown in Table 1. The sample comprised 466 male and 455 female students, with a mean age of 13.92 ± 1.00 years. There were 350 seventh-grade, 170 eighth-grade, and 401 ninth-grade students; 299 participants lived in rural areas and 622 lived in urban areas. Before questionnaire administration, all participants received written information about the study purpose, procedures, confidentiality protections, voluntary participation, and the researchers' contact details, and provided written assent.

**Table 1:** Basic information of the sample (*N* = 921).

Basic information	Category	Frequency	Percentage
Gender	Male	466	50.6%
	Female	455	49.4%
Grade	7th	350	38.0%
	8th	170	18.5%
	9th	401	43.5%
Family location	Rural	299	32.5%
	Urban	622	67.5%

The mean age was 13.92 ± 1.00 years.

### 2.2 Research methods

This analytical cross-sectional study used a structured questionnaire. Questionnaire preparation involved a literature review, initial drafting, and a pilot survey of 200 students to examine preliminary item performance, reliability, and factor structure before the main survey. The pilot participants were independent from the main survey sample and were not included in the final analyses. The final questionnaire comprised five sections.

(1) Personal background information: This section collected the participant's gender, grade level, age, and place of residence.

(2) Key competencies of physical education scale: The scale developed by Li (2022), which has subsequently been applied to Chinese secondary school samples (Wang and Yan, 2024), was used to assess KCPE. Its 24 items cover athletic ability, health behavior, and sports morality and are rated from 1 (“strongly disagree”) to 5 (“strongly agree”). Item scores were averaged within each dimension; an overall mean was used only for descriptive purposes. In the SEM, the three dimension scores served as indicators of KCPE, with higher values indicating stronger competencies. Given the multidimensional structure of KCPE and the aim of examining its overall association with the outcome variables, dimension-level means (athletic ability, health behavior, and sports morality) were used as indicators of the latent KCPE construct.

(3) Physical self-perception profile: The 30-item Physical Self-Perception Profile (PSPP) was originally developed by Fox and Corbin (1989) and adapted for Chinese samples by Xu and Yao (2001). It comprises five six-item subscales: sport competence, physical condition, body attractiveness, physical fitness, and physical self-worth. Responses range from 1 (“strongly disagree”) to 4 (“strongly agree”). Negatively worded items were recoded as *X*_R_ = 5 – *X* before subscale means were calculated, so that higher scores consistently indicated more favorable physical self-perception (PSP). Consistent with the multidimensional conceptualization of PSPP, all five domain scores were modeled as first-order indicators of the latent physical self-perception construct.

(4) Exercise-regulation scale: Exercise regulation was assessed using a 24-item measure derived from the Behavioral Regulation in Exercise Questionnaire family (Mullan et al., 1997; Markland and Tobin, 2004; Wilson et al., 2006). The measure contains four items for each of six dimensions: amotivation (AM), external regulation (ER), introjected regulation (IR), identified regulation (IDR), integrated regulation (IGR), and intrinsic motivation (IM). Items are rated from 1 (“strongly disagree”) to 5 (“strongly agree”) and averaged within each subscale, with all subscales retained in their original scoring direction. IDR, IGR, and IM served as indicators of autonomous motivation (AUT) in the hypothesized model, representing conceptually related but distinguishable forms of autonomous exercise regulation. AM, ER, and IR were retained as separate dimensions in the six-factor measurement analysis.

(5) Subjective exercise experience scale: The 12-item Subjective Exercise Experiences Scale developed by McAuley and Courneya (1994) contains four items for exercise-related positive wellbeing (PWB), four for psychological distress (PD), and four for fatigue (FAT). Items are rated from 1 (“not at all”) to 7 (“very much so”) and averaged within each dimension. Higher scores indicate more PWB, greater PD, and greater FAT on their respective scales. The three dimensions were not reverse-scored or combined into a total score. PWB was specified as the primary outcome because the study focused on positive exercise-related experiences, whereas PD and FAT were examined as complementary outcomes in supplementary models.

Table 2 summarizes the measurement instruments, response formats, scoring procedures, and roles in the analysis. The instruments were selected on the basis of their relevance to the study constructs and prior psychometric application.

**Table 2:** Scales used in this study.

Scale	Source	Items/response scale	Scoring	Role in analysis
Key competencies of physical education	Li (2022)	24 items/1-5	Means for AA, HB, and SM; overall mean descriptive only	Predictor; AA, HB, and SM as latent indicators
Physical self-perception	Xu and Yao (2001)	30 items/1-4	Negative items recoded (5 – X); five subscale means	Intermediary construct; five subscales as latent indicators
Exercise-regulation	Wilson et al. (2006)	24 items/1-5	Six separate subscale means	AUT indicated by IDR, IGR, and IM; AM, ER, and IR summarized separately
Subjective exercise experiences	McAuley and Courneya (1994)	12 items/1-7	Separate means for PWB, PD, and FAT	PWB primary outcome; PD and FAT parallel supplementary outcomes

### 2.3 Statistical analysis

Data were analyzed using IBM SPSS Statistics 27.0 and AMOS 28.0. Gender, grade level, and family location were summarized descriptively and examined in bivariate correlations. Distributional characteristics were evaluated using skewness, excess kurtosis, and normal Q–Q plots; subscale and construct means were treated as approximately continuous. Item-level confirmatory factor analyses (CFAs) tested the three-factor KCPE structure, five-factor PSPP structure, six-factor exercise-regulation structure, and three-factor SEES structure. Each item-level CFA specified correlated first-order factors, with all factor covariances freely estimated and no correlated item residuals specified. The hypothesized structural model included KCPE, physical self-perception (PSP), autonomous motivation (AUT), and positive wellbeing (PWB) as latent constructs, with their respective indicators defined by AA, HB, and SM; SC, PC, BA, PF, and PSW; IDR, IGR, and IM; and the four PWB items. Internal consistency and convergent validity were evaluated using Cronbach's α, composite reliability (CR), and average variance extracted (AVE). Discriminant validity was assessed using the Fornell–Larcker criterion (Fornell and Larcker, 1981) and the heterotrait–monotrait ratio (HTMT; Henseler et al., 2015). Measurement-model fit was evaluated using χ^2^/df, CFI, TLI, RMSEA, and SRMR. Additional fit indices (GFI, AGFI, and IFI) were also reported for the structural model. The structural model was estimated using maximum likelihood estimation. Standardized indirect associations were tested using 5,000 non-parametric bootstrap resamples and 95% percentile confidence intervals (Wen and Ye, 2014). Additional structural models were estimated by replacing PWB with PD and FAT separately as the outcome variables, while retaining the same predictor and mediator structure. Harman's single-factor test was used only as a supplementary descriptive diagnostic and was not considered sufficient to rule out common-method variance.

## 3 Results

### 3.1 Measurement properties and preliminary analyses

The item-level CFAs supported the proposed structure of each instrument. Fit indices were χ^2^/df = 1.260, CFI = 0.996, TLI = 0.995, RMSEA = 0.017, and SRMR = 0.073 for KCPE; χ^2^/df = 1.057, CFI = 0.999, TLI = 0.999, RMSEA = 0.008, and SRMR = 0.015 for PSPP; χ^2^/df = 1.093, CFI = 0.998, TLI = 0.998, RMSEA = 0.010, and SRMR = 0.011 for the six-factor exercise-regulation model; and χ^2^/df = 1.305, CFI = 0.997, TLI = 0.997, RMSEA = 0.018, and SRMR = 0.008 for the three-factor SEES model. Across the 17 first-order subscales, standardized item loadings ranged from 0.471 to 0.879, Cronbach's α from 0.811 to 0.938, CR from 0.826 to 0.954, and AVE from 0.517 to 0.706. The four constructs in the hypothesized model also showed satisfactory reliability and convergent validity (Table 3). HTMT values calculated from the indicator correlation matrix ranged from 0.340 to 0.464, below the commonly used 0.85 threshold and consistent with discriminant validity.

**Table 3:** Reliability and convergent validity of the constructs in the hypothesized model (*N* = 921).

Construct	Indicators	Loading range	α	CR	AVE	√AVE
KCPE	AA, HB, SM	0.880–0.921	0.927	0.927	0.810	0.900
PSP	SC, PC, BA, PF, PSW	0.868–0.905	0.950	0.950	0.791	0.889
AUT	IDR, IGR, IM	0.873–0.890	0.913	0.914	0.780	0.883
PWB	PWB1–PWB4	0.788–0.805	0.875	0.875	0.636	0.798

Standardized loadings are from the four-construct measurement model. α, Cronbach's alpha; AUT, autonomous motivation; AVE, average variance extracted; CR, composite reliability; KCPE, key competencies of physical education; PSP, physical self-perception; PWB, exercise-related positive wellbeing.

Across the 17 first-order dimension scores, absolute skewness ranged from 0.021 to 0.495 and absolute excess kurtosis from 0.061 to 0.459. Q–Q plots were approximately linear across the central quantiles, with modest tail departures consistent with bounded Likert-type composite scores (Figures 1, 2). These results did not indicate severe univariate non-normality and were considered adequate for maximum-likelihood estimation given the large sample.

**Figure 1:**
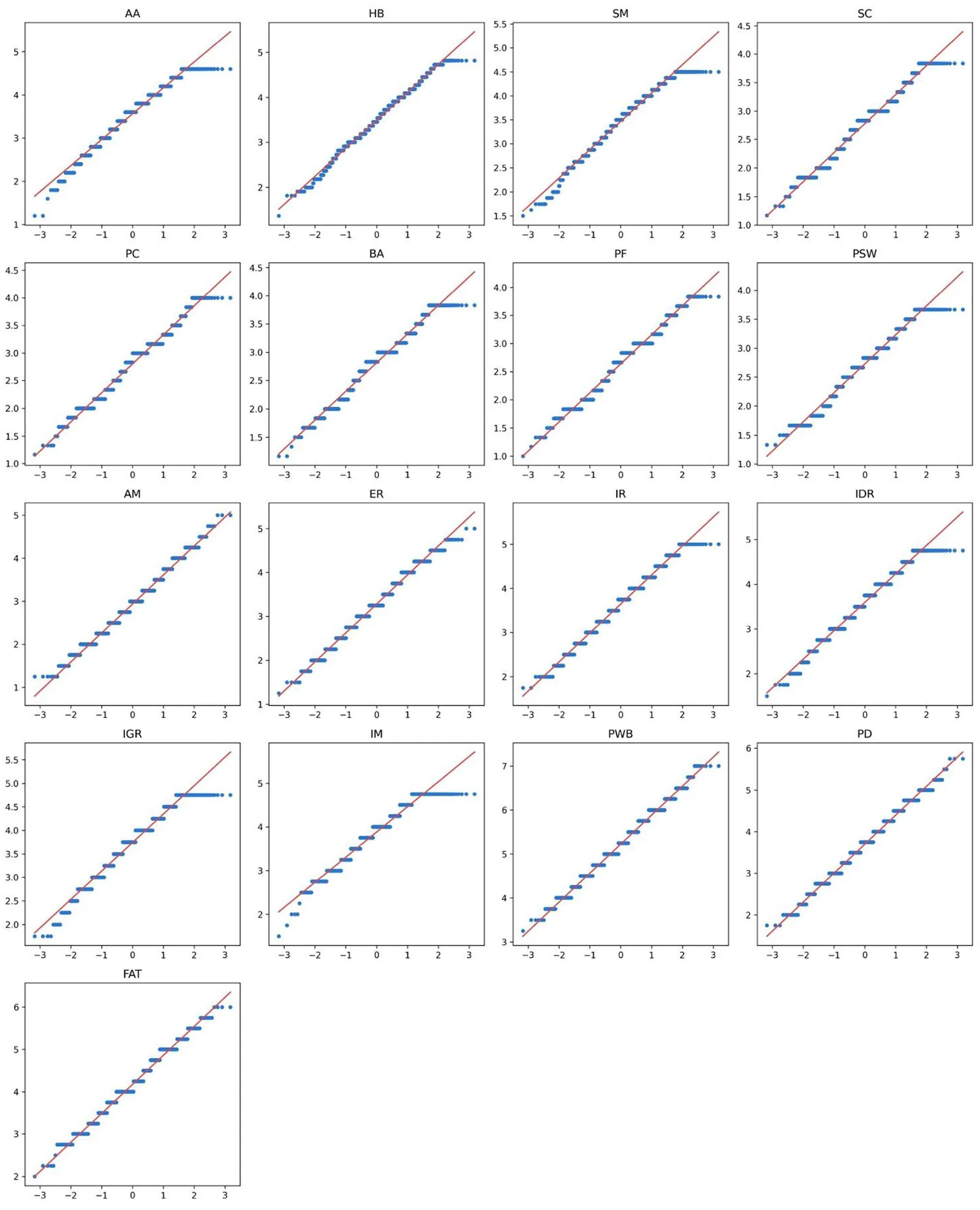
Normal *Q–Q* plots for the first-order dimension scores.

**Figure 2:**
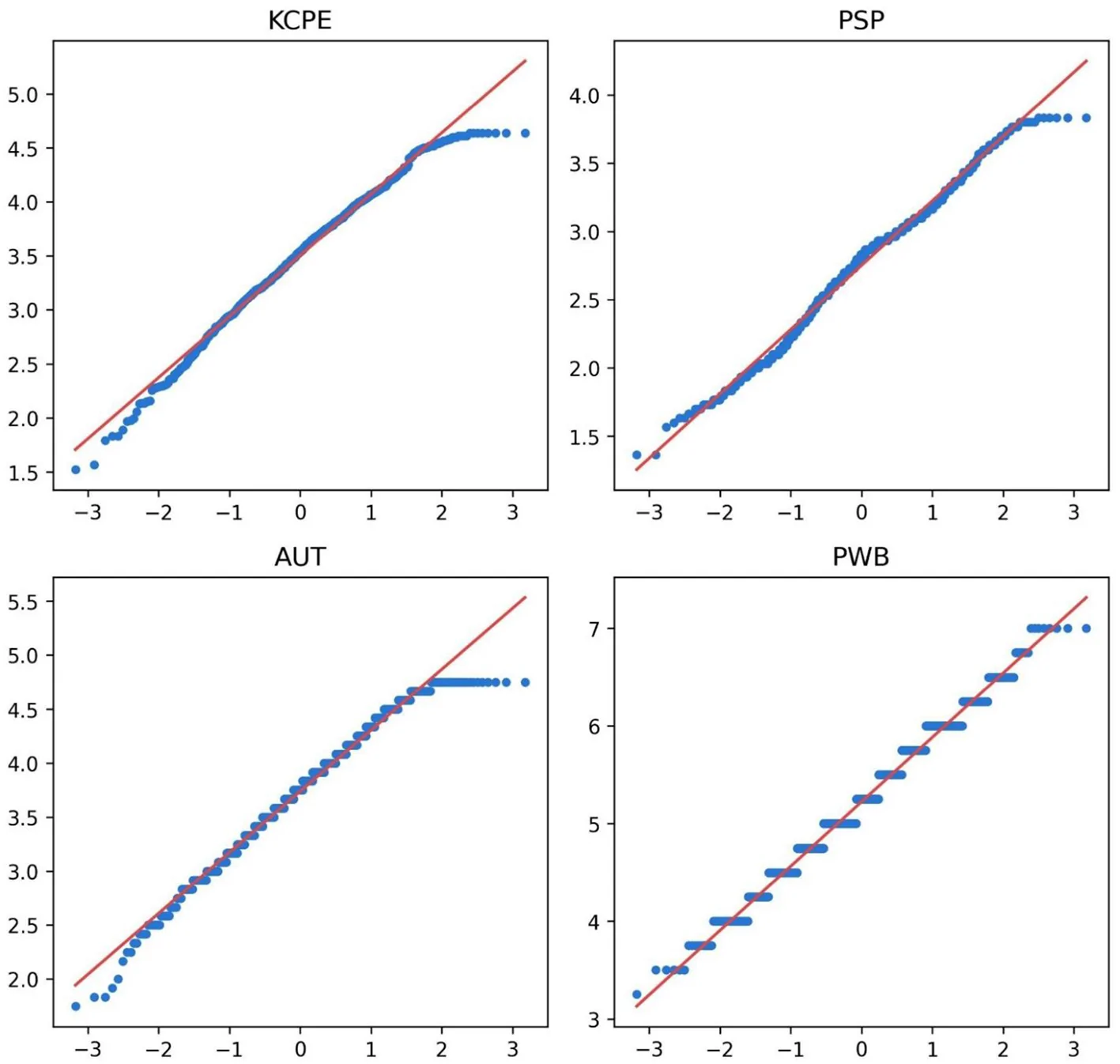
Normal *Q–Q* plots for the four construct scores used in the hypothesized model.

Harman's single-factor test yielded 17 factors with eigenvalues greater than 1, and the first unrotated factor accounted for 24.543% of the total variance. However, this result should be interpreted only as a limited descriptive diagnostic and does not exclude the possibility of common-method variance.

### 3.2 Descriptive and correlation analyses

Descriptive statistics and latent correlations for the four constructs in the hypothesized model are shown in Table 4. KCPE was positively correlated with PSP (*r* = 0.390), AUT (*r* = 0.462), and PWB (*r* = 0.386); PSP was correlated with AUT (*r* = 0.417) and PWB (*r* = 0.338); and AUT was correlated with PWB (*r* = 0.455; all *p* < 0.001). For each construct, the square root of AVE (0.798–0.900) exceeded its correlations with the other constructs. At the observed-score level, AM (*M* = 2.936, *SD* = 0.677), ER (*M* = 3.280, *SD* = 0.664), and IR (*M* = 3.645, *SD* = 0.661) were not significantly correlated with KCPE, PSP, AUT, or PWB (|r| ≤ 0.047). PD (*M* = 3.698, *SD* = 0.699) and FAT (M = 4.175, SD = 0.691) likewise showed no significant correlations with the four main constructs (|r| ≤ 0.030). Among the examined demographic characteristics, only family location showed a statistically significant association (*p* = 0.023) with autonomous motivation, while gender and grade level were not significantly associated with the study variables. Given the limited magnitude of demographic associations and the theoretical focus of the hypothesized psychological model, demographic variables were not included as covariates in the structural model.

**Table 4:** Descriptive statistics and latent correlations among constructs in the hypothesized model (*N* = 921).

Construct	M ±SD	KCPE	PSP	AUT	PWB
KCPE	3.506 ± 0.569	0.900			
PSP	2.753 ± 0.473	0.390	0.889		
AUT	3.739 ± 0.568	0.462	0.417	0.883	
PWB	5.223 ± 0.663	0.386	0.338	0.455	0.798

Values on the diagonal are the square roots of AVE; values below the diagonal are standardized latent correlations, all *p* < 0.001. M ± SD values are based on observed composite scores.

### 3.3 Structural model and indirect associations

The hypothesized structural model showed good fit, χ^2^(84) = 117.525, *p* = 0.009, χ^2^/df = 1.399, CFI = 0.997, GFI = 0.983, AGFI = 0.976, TLI = 0.996, IFI = 0.997, RMSEA = 0.021, 90% CI [0.011, 0.029], and SRMR = 0.013 (Figure 3). Standardized path coefficients indicated positive associations from KCPE to PSP (β = 0.390), AUT (β = 0.353), and PWB (β = 0.190); from PSP to AUT (β = 0.280) and PWB (β = 0.134); and from AUT to PWB (β = 0.312; all *p* < 0.001). The model explained 15.2% of the variance in PSP, 28.0% in AUT, and 26.1% in PWB.

**Figure 3:**
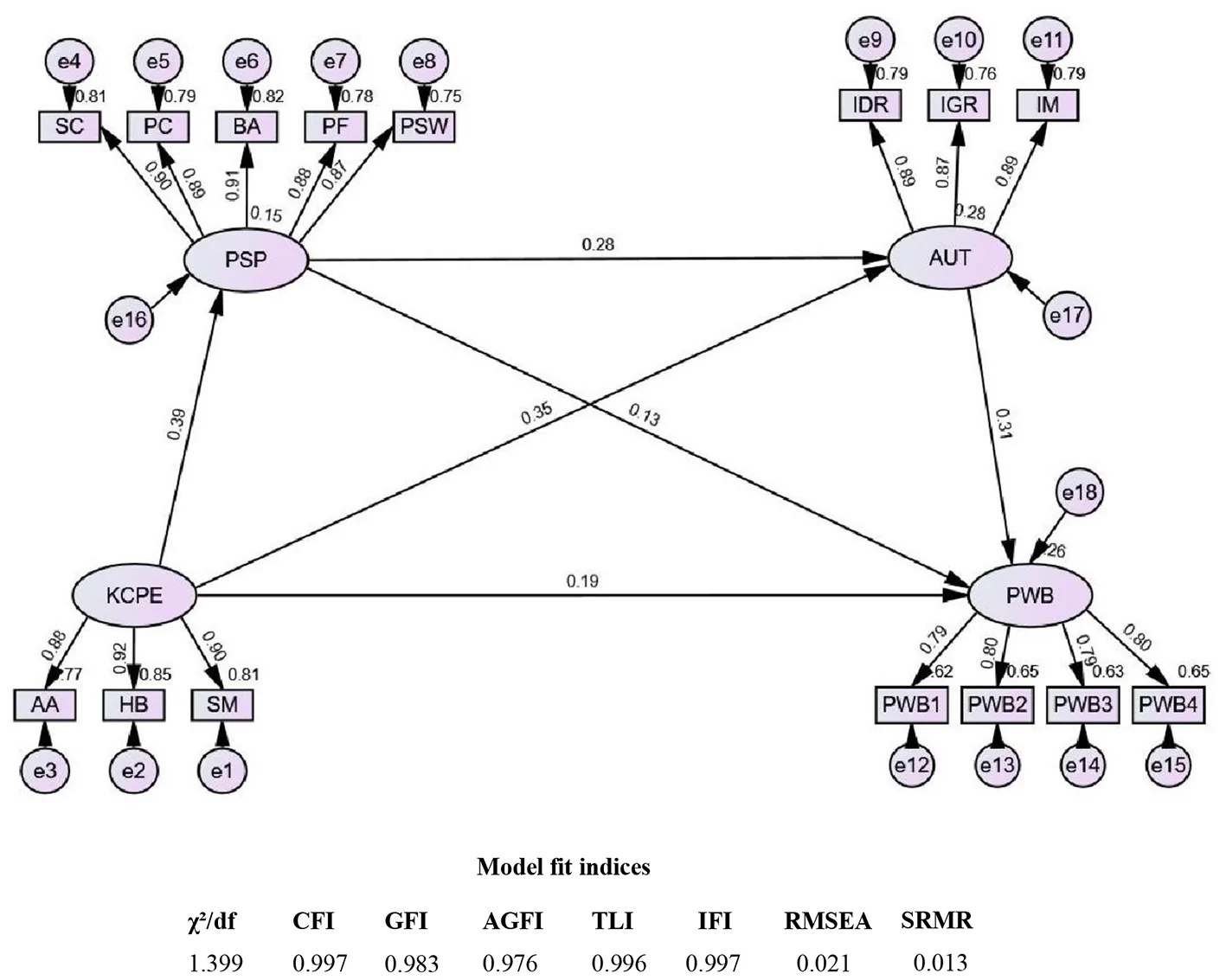
Structural equation model for KCPE, physical self-perception, autonomous motivation, and exercise-related positive wellbeing. Values are standardized coefficients. AA, athletic ability; HB, health behavior; SM, sports morality; SC, sport competence; PC, physical condition; BA, body attractiveness; PF, physical fitness; PSW, physical self-worth; IDR, identified regulation; IGR, integrated regulation; IM, intrinsic motivation; PWB1–PWB4, items representing exercise-related positive wellbeing. All reported structural paths were statistically significant at *p* < 0.001.

Bootstrap results are presented in Table 5. The standardized indirect association through PSP was 0.052 [95% CI (0.024, 0.081)], the indirect association through AUT was 0.110 [95% CI (0.078, 0.146)], and the specified serial indirect pathway through PSP and AUT was 0.034 [95% CI (0.022, 0.048)]. None of the confidence intervals included zero; thus, H2–H4 received statistical support within the specified model. The total indirect association was 0.196, the direct association was 0.190, and the total association was 0.386, indicating that both direct and indirect associations contributed to the observed total association. In the parallel supplementary models, the structural paths to PD (KCPE → PD β = 0.061; PSP → PD β = −0.063; AUT → PD β = −0.009) and FAT (KCPE → FAT β = −0.008; PSP → FAT β = −0.004; AUT → FAT β = 0.003) were not statistically significant. Accordingly, the statistically significant outcome-directed associations in these data were more evident for PWB than for the negative dimensions of subjective exercise experience.

**Table 5:** Standardized direct and indirect associations between KCPE and exercise-related positive wellbeing (*N* = 921).

Association type	Association pathway	Standardized association	Bootstrap SE	Bootstrap 95% CI
Direct association	KCPE → PWB	0.190	0.038	[0.115, 0.265]
Indirect associations	KCPE → PSP → PWB	0.052	0.015	[0.024, 0.081]
	KCPE → AUT → PWB	0.110	0.017	[0.078, 0.146]
	KCPE → PSP → AUT → PWB	0.034	0.007	[0.022, 0.048]
Total indirect association		0.196	0.022	[0.155, 0.239]

## 4 Discussion

This study examined associations between KCPE and exercise-related positive wellbeing among secondary school students surveyed in selected districts of Beijing. All hypothesized direct and indirect associations involving physical self-perception and autonomous motivation were statistically significant for exercise-related positive wellbeing. In contrast, when psychological distress and fatigue were examined as alternative dimensions of exercise experience, the corresponding associations were small and not statistically significant. Because all focal variables were measured at the same time point, the directional arrows in the SEM represent probabilistic directional associations under the theoretically specified model, not observed temporal processes or causal sequences.

### 4.1 The association between key competencies of physical education and exercise-related positive wellbeing

Although related to physical literacy, KCPE represents a curriculum-specific competency framework; therefore, findings from physical literacy research provide contextual rather than direct evidence for interpreting the present results. The positive association between KCPE and exercise-related positive wellbeing was consistent with H1 and with earlier work linking competence-related constructs to favorable affective experiences during physical activity. Blain et al. (2021) reported an association between physical literacy and subjective wellbeing among early adolescents, and Melby et al. (2022) found positive cross-sectional associations between physical literacy and wellbeing in Danish adolescents. In the present study, students who reported stronger athletic ability, health behavior, and sports morality also tended to report more favorable feelings during exercise. This finding concerns situational exercise-related wellbeing and should not be interpreted as evidence regarding general mental health.

### 4.2 The indirect association through physical self-perception

The estimated indirect association through physical self-perception was statistically significant, supporting H2 within the specified cross-sectional model. Students reporting stronger KCPE also tended to report more favorable perceptions of their sport competence, physical condition, body attractiveness, physical fitness, and physical self-worth, and these perceptions were associated with greater exercise-related positive wellbeing. This pattern is consistent with theoretical links among competence experiences, physical self-evaluation, and affect during exercise (White et al., 2021). It also supports treating physical self-perception as a multidimensional profile in which physical self-worth is one component rather than a label for the entire construct. Importantly, this perspective highlights the need to consider students with less favorable physical self-perceptions in physical education contexts. Students who report lower perceived competence or physical self-worth may be more vulnerable to reduced confidence, avoidance of participation, and less favorable exercise experiences. Therefore, physical education practices emphasizing progressive mastery, individual improvement, and supportive feedback may be particularly appropriate for students with less favorable physical self-perceptions.

### 4.3 The indirect association involving autonomous motivation

Among the three specific indirect associations, the association through autonomous motivation was numerically the largest and was statistically significant, consistent with H3. Autonomous motivation was represented by identified regulation, integrated regulation, and intrinsic motivation. This result accords with self-determination theory research indicating that need-supportive physical education and meaningful, self-endorsed reasons for participation are associated with more favorable motivational and affective outcomes (Vasconcellos et al., 2020; Manninen et al., 2022; Qi et al., 2024).

### 4.4 The modeled serial indirect association involving physical self-perception and autonomous motivation

The specified serial indirect pathway involving physical self-perception and autonomous motivation was statistically significant, supporting H4 within the specified model. Stronger KCPE was associated with more favorable physical self-perception; physical self-perception was associated with more autonomous reasons for exercise; and autonomous motivation was associated with greater exercise-related positive wellbeing. This pattern is consistent with adolescent research showing associations between physical self-concept and autonomous motivation, including studies using structural equation modeling approaches (Pulido et al., 2021; Núñez et al., 2021; Valero-Valenzuela et al., 2021). The proposed order is theoretically plausible, but its temporal sequence cannot be determined from variables measured concurrently.

Taken together, the observed direct and indirect association pattern was more evident for exercise-related positive wellbeing, whereas the corresponding associations involving psychological distress and fatigue were not statistically significant. These findings support the view that the three SEES dimensions reflect distinct aspects of exercise experience rather than a uniform outcome domain. The interpretation of the structural model should also consider the characteristics of the measurement approach. Specifically, KCPE, physical self-perception, and autonomous motivation were represented by manifest dimension scores rather than item-level indicators, whereas positive wellbeing was represented by its four items. This aggregation may reduce item-specific residual complexity and contribute to the relatively high model fit indices (e.g., CFI = 0.997). Therefore, the excellent fit should not be interpreted as evidence of perfect model validity; rather, it indicates that the specified model adequately reproduced the observed covariance structure among the selected indicators in this sample.

### 4.5 Practical implications for physical education practice

Although the present study did not test intervention effects, its findings suggest that competency-oriented physical education should attend to both the development of integrated competencies and the quality of students' exercise-related experiences. Consistent with self-determination theory, autonomy- and competence-supportive environments are associated with more favorable motivational and affective outcomes (White et al., 2021; Vasconcellos et al., 2020). In Beijing secondary-school settings, teacher training could focus on integrating athletic ability, health behavior, and sports morality into everyday teaching rather than treating them as separate instructional targets. Teachers could organize learning tasks progressively and adjust their difficulty to students' existing abilities, allowing those with less favorable physical self-perceptions, particularly lower perceived competence or physical self-worth, to experience attainable improvement and more positive participation. Skill practice could also be accompanied by guidance on exercise methods, safety, recovery, and regular participation, while cooperation, competition, rule observance, and shared responsibility could provide meaningful contexts for cultivating sports morality.

Students requiring additional support could be identified through routine observation and private communication, thereby avoiding public comparison or labeling. For these students, early support could emphasize scaffolded success and supportive feedback. As their sense of competence develops, teachers could gradually introduce greater choice, personal goal setting, and opportunities to develop more self-endorsed reasons for exercise. This staged approach is consistent with the modeled association pattern involving physical self-perception and autonomous motivation. However, because these variables were measured concurrently, the present study did not establish the temporal order or instructional effectiveness of this sequence. Beyond classroom instruction, students' exercise experiences are shaped by interacting individual, interpersonal, and educational influences across settings. Although recent DE-PASS syntheses address physical activity-related outcomes more broadly, they highlight the importance of considering determinants operating across multiple contexts (Ling et al., 2024; Khudair et al., 2025). KCPE development may therefore be better supported when curriculum design, teaching practices, peer interactions, and assessment approaches work together to promote students' engagement and positive exercise-related experiences. Longitudinal and intervention studies are needed to evaluate the proposed instructional sequence and its applicability across different school contexts.

### 4.6 Limitations

This study has several limitations. First, the cross-sectional design cannot establish temporal order; therefore, the indirect associations should not be interpreted as causal mediation. Second, all focal variables were assessed using concurrent self-report questionnaires, so common-method variance may have contributed to the observed associations; the Harman single-factor result cannot rule out this possibility. Future studies incorporating multi-source assessments or longitudinal designs may strengthen the evidence. Third, although participants were recruited from 12 secondary schools across 8 districts and 36 classes, school- and class-level identifiers were unavailable. Accordingly, the SEM used conventional maximum-likelihood standard errors and did not adjust for clustering at the school or class level. If responses were correlated within schools or classes, the reported standard errors may therefore be underestimated and the statistical significance of some paths overstated. Future studies should retain school- and class-level identifiers and use cluster-robust or multilevel estimation. Finally, the primary structural findings concerned exercise-related positive wellbeing; the supplementary psychological distress and fatigue models require further examination before broader conclusions are made regarding other dimensions of subjective exercise experience.

## 5 Conclusion

Among secondary school students surveyed in selected districts of Beijing, KCPE was positively associated with exercise-related positive wellbeing. Physical self-perception and autonomous motivation were associated with statistically significant specific indirect pathways, including the specified serial indirect pathway. By contrast, the parallel models for psychological distress and fatigue showed no significant paths to the outcome variables. These findings support the proposed cross-sectional association pattern and provide a basis for longitudinal and intervention research capable of testing temporal ordering and potential causal relationships.

## Data Availability

The original contributions presented in the study are included in the article. Further inquiries can be directed to the corresponding author.

## Footnotes

1 World Health Organization (2025). Mental health of adolescents. https://www.who.int/news-room/fact-sheets/detail/adolescent-mental-health.
